# Lucretia W. McClure, AHIP, FMLA

**DOI:** 10.5195/jmla.2020.908

**Published:** 2020-04-01

**Authors:** Judith Messerle

**Affiliations:** Director (Retired), Countway Library of Medicine, Harvard Medical School, Boston, MA, jmesserle@frontiernet.net

## Abstract

With the passing of Lucretia W. McClure, AHIP, FMLA, on October 17, 2019, the Medical Library Association, the library profession, and the field of medical history lost an important friend and colleague. She was a role model for many and a bright light for the association.

The North Star is a beacon for sailors navigating heavy seas, a fixed light that helps guide them to their ultimate destination. On October 17, 2019, the medical library profession lost its North Star when Lucretia W. McClure, AHIP, FMLA, passed peacefully from this earth in her hometown of Rochester, New York. For more than half a century, Lucretia helped lead us through continuous change, educated us, cheered us on, and moved us forward. She would not have wanted to accept credit for it, but her record illustrates the breadth of her role in the profession, in the Medical Library Association (MLA), and in the history of medicine.

Born in Denver, Colorado, on January 2, 1925, Lucretia was the middle child between older brother John and sister Margaret. Hers was a family that enjoyed books, and she became a frequent user of the public libraries in Denver. However, librarianship was not Lucretia’s first choice for a profession. She graduated from the University of Missouri with a degree in journalism in 1945. This career was not what she anticipated, so she elected to marry and raise two boys, Paul and John. She developed an interest in librarianship, and in 1964 with the support of her husband Arnold, she took her two sons to Colorado and earned a master of arts degree (MA) in librarianship from the University of Denver.

She and her children reunited with her husband when she took a cataloging position at the Edward G. Miner Library, University of Rochester Medical Center. Lucretia loved the medical literature, the precision of librarianship, and the intellectual challenges of cataloging and found great joy in the provision of reference service. These passions, refined over the decades, made her the quintessential medical librarian. She was beloved by students, Nobel laureates, deans, and anyone else who sought her help. As she moved up through the ranks to become library director, she was a mentor to all. She retired for the first time in 1993.

**Figure:**
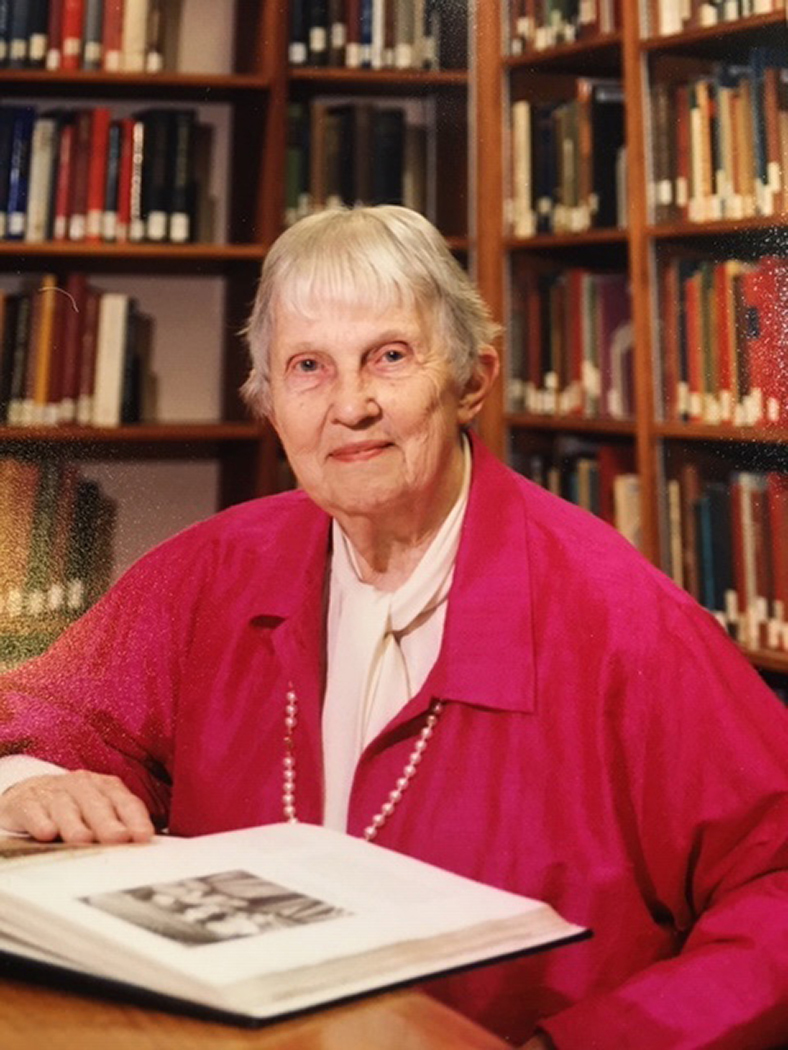


Lucretia’s entry into the profession came at a time of major transition. She learned her library skills before the advent of the copy machine and computerized records. The Biomedical Communications Network (BCN) provided the first online searches of the *Index Medicus*, and Lucretia was on its board from the outset. She saw the creation of the National Library of Medicine’s (NLM’s) controlled vocabulary thesaurus Medical Subject Headings (MeSH). She had a front row seat for the development of OCLC at University of Rochester. She saw the Medical Library Assistance Act of 1965 revitalize post-war medical library funding and training, and the implementations of the bibliographic retrieval system AIM-TWX, MEDLINE, Grateful Med, and PubMed. It was a heady time to be a medical librarian.

Following her first retirement, she received many consulting requests. A call in 1996 to evaluate the rare book collection at Harvard Medical School’s Countway Library turned into a full-time position as special assistant to the director. She flew every Sunday to Boston and lent her expertise on many fronts including the reference desk, the rare book department, and the reorganization of 600,000 volumes during a major renovation. She came to work early and left late. On Thursday nights, she flew home to Rochester for the weekend. She retired again in 2011 at 86.

When she moved to a Rochester retirement community, Valley Manor, in 2012, she became director of their library. After she led a major renovation, the library was named in her honor in 2017. No wonder her colleagues affectionately called her the “Energizer Bunny.” She held the position at Valley Manor until her death at ninety-four.

This remarkable record is paralleled only by her commitment to collegial work in library associations. Lucretia became a member of MLA in 1964. There, she met lifelong friends and admirers and established her reputation for engagement and hard work. Lucretia’s work ethic resulted in many MLA assignments, from committee chairs to the presidency (1990/91). Colleagues trusted that if Lucretia agreed to do something, the product would be pristine.

Her oral history notes that she would always say yes to invitations that opened doors to new ideas and people, and as a result, she was often asked to serve. It was one such request that led to her appointment as MLA’s copyright referent in 1983. Another request led to her appointment as MLA parliamentarian, a post she held for many years.

Lucretia was also well known at NLM. She served on panels, consulted, and worked with interns. From a position on the Friends of the National Library of Medicine Board to chair of the Michael E. DeBakey Medical Library Services Outreach Award Committee, she was a regular visitor and fully accepted as NLM family.

Her love of medical history positioned Lucretia as a charter member of the Archivists and Librarians in the History of the Health Sciences (ALHHS), an offshoot of the American Association for the History of Medicine (AAHM). She attended their annual meetings and wrote frequently for their newsletter, *The Watermark.*

Her excitement about library services for the public led to other appointments as Lucretia lobbied for integration of resources across libraries. She served as a member of the New York State Regents Advisory Council on Libraries and as president of the Rochester Regional Library Council Board.

Those who participated in one of the thirty-five “Management of Reference” classes that she taught around the country and abroad still remember the lessons they learned. For these hundreds of students, it was often the introduction to Lucretia herself that was the lifelong touchstone.

Given her love of language, she was always writing. At the Countway Library and Valley Manor, she edited and wrote the libraries’ monthly newsletters and produced a column, “Honoring Our Past,” for the *MLA News.* Her many professional publications focused on subjects dear to her heart: education of medical librarians, medical history, copyright, and library practices. She traveled around the country speaking on these topics, encouraging active participation in the profession. Her grandchildren were the beneficiaries of her delightful children’s tales, which she wrote in Boston and delivered to Rochester on weekends home.

Juggling all these responsibilities would exhaust the most dedicated, organized person. Her desk overflowed with papers; her carts were filled with books. She was always researching and reading, but somehow Lucretia always had time for more. She had time for people.

From her early days as a librarian, she was always looking to engage intellectually and relate to others. Lucretia was the perfect MLA emissary to greet new medical librarians at the annual meeting welcome session. She made them feel that they could make a difference and that their neighbors at the welcome session tables could become their friends for life. “People count,” she would say. She opened the arms of the association and took in new members. She never spoke negatively of a colleague or an idea. One could share a confidence with absolute trust that the conversation would stay only with her. Some who worked closely with her would say that she was their “second mother.”

Getting together with Lucretia at meetings was a ritual for many. To get a private word of advice, support, and confirmation from her was important. Her dinner dates, break times, and side meetings filled up quickly. She enjoyed the give and take, the conversations, and the remembrances. Being with Lucretia was joyful. She was witty and often profound. She loved the color purple, sensible shoes, and a good glass of pinot grigio. When others were done for the day, Lucretia was literally ready to dance.

Recognition of Lucretia’s contributions came steadily throughout her career, even though she was never one to seek the limelight. She received all of MLA’s highest honors, including the Marcia C. Noyes Award, the Janet Doe Lectureship, and the MLA President’s Award. She was the first recipient of the Lucretia W. McClure MLA Excellence in Education Award, created in her honor. A “Lucretia McClure Day” was declared at the 2013 MLA annual meeting in Boston. Among many other awards, she was declared “A Living Treasure” at the Upstate New York and Ontario Chapter 2011 annual meeting.

Lucretia cared passionately about the education of librarians, urging greater MLA influence in library schools. She promoted increased interaction with other medical professional associations. She worried about medical students’ heavy reliance on online information. She was concerned about the diminished presence of history in medical education. She advocated for changes in these areas, often writing to new MLA presidents-elect to push for her ideas.

For us, for MLA, and for the medical library profession, Lucretia McClure was our North Star. Her ethics and humanity; her love of literature, writing, and the medical library field; and most of all, her respect for and appreciation of people will keep us pointing toward her guiding light for years to come.

Lucretia will be missed by her beloved family: sons John and his wife Cindy, and Paul and his wife Lauren; grandsons Ben, Eric, Jonathan, and Andrew; sister Margaret; and multitudes of friends and colleagues.

**Judith Messerle, AHIP, FMLA**, jmesserle@frontiernet.net, Director (Retired), Countway Library of Medicine, Harvard Medical School, Boston, MA

